# Protein pI and Intracellular Localization

**DOI:** 10.3389/fmolb.2021.775736

**Published:** 2021-11-29

**Authors:** Alexander A. Tokmakov, Atsushi Kurotani, Ken-Ichi Sato

**Affiliations:** ^1^ Department of Genetic Engineering, Faculty of Biology-Oriented Science and Technology, Kindai University, Wakayama, Japan; ^2^ Center for Sustainable Resource Science, RIKEN Yokohama Institute, Yokohama, Japan; ^3^ Laboratory of Cell Signaling and Development, Faculty of Life Sciences, Kyoto Sangyo University, Kyoto, Japan

**Keywords:** protein pI, proteome-wide analysis, multimodality, subcellular localization, local environment

## Abstract

The protein isoelectric point (pI) can be calculated from an amino acid sequence using computational analysis in a good agreement with experimental data. Availability of whole-genome sequences empowers comparative studies of proteome-wide pI distributions. It was found that the whole-proteome distributions of protein pI values are multimodal in different species. It was further hypothesized that the observed multimodality is associated with subcellular localization-specific differences in local pI distributions. Here, we overview the multimodality of proteome-wide pI distributions in different organisms focusing on the relationships between protein pI and subcellular localization. We also discuss the probable factors responsible for variation of the intracellular localization-specific pI profiles.

## Introduction

The isoelectric point (pI) of a protein is defined as the pH at which the net charge of a protein molecule is zero. Accordingly, proteins are positively charged at a pH below their pI and negatively charged at a pH above their pI. The protein pI varies greatly from extremely acidic to highly alkaline values ranging from about 4.0 to 12.0. Hence, pI values have long been used to distinguish between proteins in methods for protein isolation, separation, purification, crystallization, etc. Amino acid composition of a protein sequence primarily defines its pI, based on the combination of dissociation constant (pKa) values of the constituent amino acids. Out of twenty common amino acids, two amino acids, aspartic acid, and glutamic acid, are negatively charged and three amino acids, lysine, arginine, and histidine, are positively charged at the neutral pH, as defined by their pKa values. Thus, the integral property of a protein, such as protein pI, was supposed to result from discrete local acidic and basic pKas of amino acid side chains. It was demonstrated that the protein pI can be estimated based on a polypeptide sequence in close agreement with experimentally determined pI values (Sillero and Ribeiro, 1989), and the focusing positions of proteins in immobilized pH gradients and two-dimentional gels can be reliably predicted from their amino acid composition (Bjellqvist et al., 1993; Bjellqvist et al., 1994; Link et al., 1997). Notably, three-dimensional structure and pH of surrounding environment can influence ionizable groups and affect the net charge on the molecule significantly (Russell and Warshel, 1985).

Various calculative algorithms have been developed for estimating protein pIs in agreement with experiments regardless of structural aspect (Gasteiger et al., 2003; Cargile et al., 2004; Gauci et al., 2008; Maldonado et al., 2010; Audain et al., 2016). Some methods take into account the effect of the amino acids residues adjacent to the charged residues, such as aspartate and glutamate (Cargile et al., 2008), effects of posttranslational modifications, such as phosphorylation and N-terminal acetylation (Gauci et al., 2008), or effects of the presence of polyelectrolyte chains around proteins (Srivastava et al., 2017). In addition, the experimentally observed protein pI values were summarized in the experimental databases (Hoogland et al., 2004; Bunkute et al., 2015). Also, a database of protein pIs that were predicted using multiple available methods has been presented (Kozlowski, 2017).

Thus, protein pI is an integral property of a protein molecule fundamentally important for its characterization. The great variation of protein pI values brings about the question about the cause of this variation. Availability of whole-genome sequences allows comparative and evolutionary studies of proteome-wide pI distributions in different organisms. These studies have revealed important universal features of the whole proteome pI distributions providing insights into spatial organization of cellular proteomes. The localization- and function-specific differences in subcellular pI distributions have been disclosed. Our present paper overviews proteome-wide pI distributions focusing on the relationships between protein pI and subcellular localization.

## Intrinsic Bimodality of Protein pI Distributions

**FIGURE 1:**
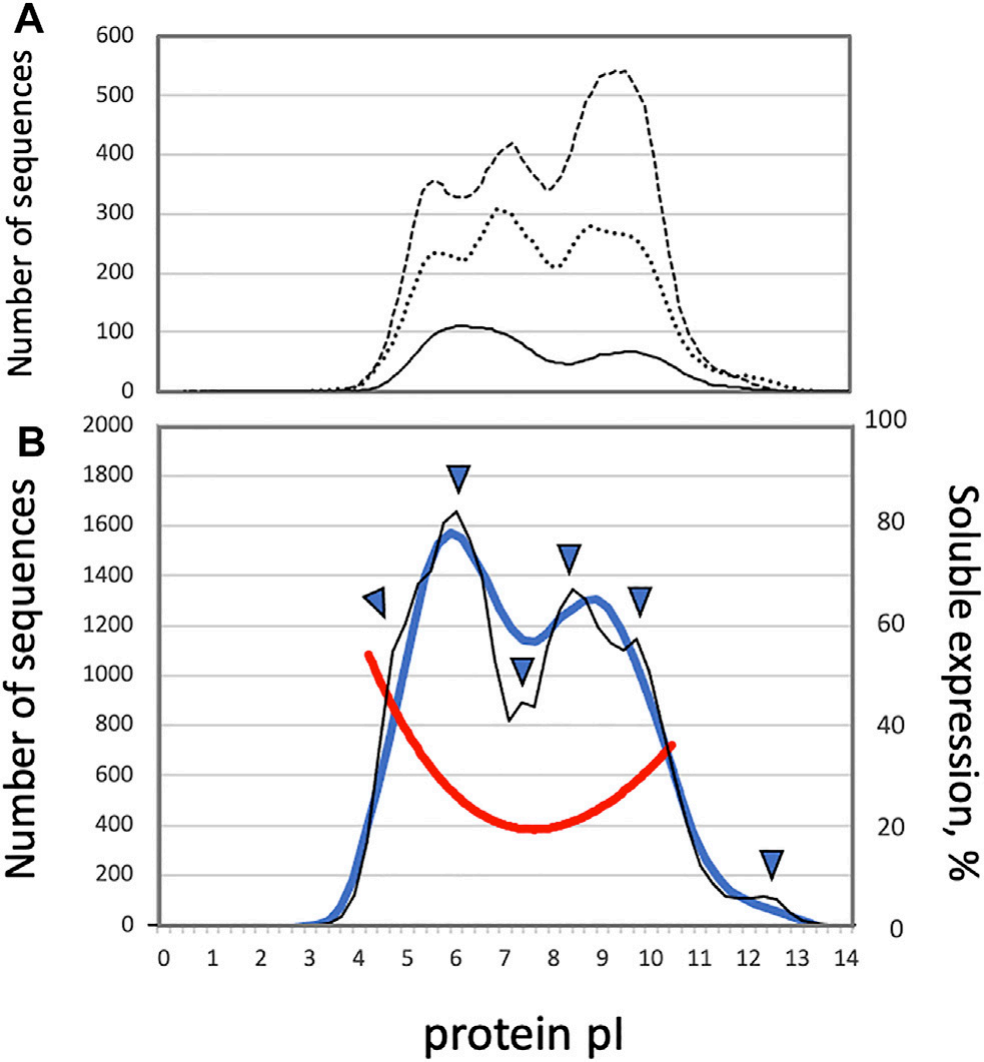
**(A)** Smoothened distributions of protein pI in the proteomes of *Escherichia coli* (solid line), *Drosophila melanogaster* (dotted line) and *Caenorhabditis elegans* (dashed line), as based on the histogram data presented by Schwartz et al. (Schwartz et al., 2001) **(B)** Distributions of protein pI and solubility in the human proteome. The thick blue line and thin black line show smoothened and un-smoothened whole-proteome distributions of protein pI values, respectively. The red line presents solubility of cell free-expressed human proteins. Arrowheads indicate the positions of shoulders and sub-peaks observed in the whole-proteome pI distribution.

The early studies of proteome-wide pI distributions demonstrated that they are bimodal, with distinct acidic and alkaline peaks, in several bacterial strains (Blattner et al., 1997; Urquhart et al., 1997; VanBogelen et al., 1999). The two major protein clusters, centering around pI 5.0 and pI 9.0, were observed in full proteomes of bacteria and archaea (Schwartz et al., 2001; Figure 1A). It was suggested that the low abundance of sequences with unbiased pIs curtails protein precipitation at a near-neutral physiological pH. Indeed, the pI value affects solubility of a protein molecule at a given pH. Proteins display the least solubility in water-based solutions at the pH that corresponds to their pI, often resulting in protein aggregation (Arakawa and Timasheff, 1985). It was demonstrated experimentally, using cell-free protein synthesis, that protein solubility positively correlates with the content of charged residues in the expressed proteins, and the proteins with pI 7.0–7.5 have the lowest rate of soluble expression (Kurotani et al., 2010; Tokmakov et al., 2014; Figure 1B). On the other hand, the ratio of high to low cell-free expression levels was found to be stable in the wide range of pI values (Tokmakov et al., 2014), suggesting the absence of correlation between protein pI and expression level. Several studies proposed that the pI multimodality observed in different proteomes could be rooted in discrete pKa values for different amino acids (Weiller et al., 2004; Wu et al., 2006; Garcia-Moreno, 2009). Importantly, it was found that the pI distributions of cytosolic and integral membrane proteins corresponded to the two modes observed in the whole-proteome pI distributions. Cytoplasmic proteins clustered at pI 5.0 to 6.0, and integral membrane proteins exhibited a distinct clustering at pI 8.5 to 9.0 (Schwartz et al., 2001). Also, investigation of complete predicted proteomes using theoretical 2D gels (MW vs pI) indicated that the membrane proteomes are generally more alkaline than the non-membrane ones (Knight et al., 2004). The alkaline bias of the membrane proteins was attributed to the fact that biomembranes generally bear a negative charge due to the presence of negatively charged phospholipids, thus the positive charge of basic proteins at normal pH would promote favorable electrostatic interactions stabilizing the proteins in the membranes (Schwartz et al., 2001). These data strongly suggested a link between the whole-proteome pI distributions and subcellular localization.

## Common Multimodality of pI Distributions

Further investigations revealed that the protein pI profiles are trimodal in many eukaryotic proteomes (Figure 1A), and the presence of the third peak was linked to the appearance of the nuclear compartment in eukaryotes. Nuclear proteins were revealed to have a wide distribution varying from pI 4.5 to pI 10.0 (Schwartz et al., 2001). Several additional modes, such as a minor peak at the pI above 11.0, were distinguished in the whole-proteome pI distributions of eukaryotic proteins (Wu et al., 2006; Carugo, 2007), further suggesting the presence of divergent subcellular protein pI profiles. Markedly, the trimodality of proteome-wide pI distributions is not conserved across eukaryotic species. Although trimodal distributions of protein pI have been observed in some proteomes of eukaryotic species, such as *Saccharomyces cerevisiae, Caenorhabditis elegans, and Drosophila melanogaster* (Schwartz et al., 2001; Figure 1A), bimodal distributions of pI were witnessed in the proteomes of human, mouse, and malaria plasmodium (Medjahed et al., 2003). In addition, contrary to the earlier study, it was reported that the global pI distribution of *C. elegans* and *S. cerevisiae* proteins are bimodal (Medjahed et al., 2003; Ho et al., 2006), as explained by difference in the algorithms employed for calculation of protein pI. Also, our recent study demonstrated that the profile of protein pI values determined for the complete human proteome is essentially bimodal with the major acidic and alkaline peaks at around pI 6.0 and pI 8.25 (Kurotani et al., 2019, Figure 1B). Notably, the two major peaks of the pI distribution are not Gaussian and not well-resolved, leaving open the possibility that the broad modality corresponding to nuclear proteins may be obscured by the two major overlapping peaks. Moreover, the distribution of human proteins displayed some additional statistical features, such as minor peaks and peak shoulders (Kurotani et al., 2019, Figure 1B). Protein localization patterns were further analyzed throughout the whole-proteome pI distribution, and it was found that the observed major and minor peaks of the distribution were associated with specific subcellular localizations (Kurotani et al., 2019).

## Adaptation of pI Patterns to Environmental Constraints and Evolutionary Aspects

The average proteome pI and relative abundance of the acidic and alkaline peaks in bimodal pI distributions were analyzed in connection with organism taxonomy and environment. It was reported that proteome pI adapts to the conditions of bacterial growth; a significant positive correlation was observed between predicted proteome distributions on the theoretical 2D gels (MW vs pI) and the Biolog profile, a measure associated with ecological niche (Knight et al., 2004). It was noted that smaller proteomes of intracellular parasites are more alkaline because of their adaptation to elevated host pH (Knight et al., 2004). It was also reported that, proteome pI adjusts to high-temperature environmental conditions of *Thermoplasma volcanium* growth (Kawashima et al., 2000). A later bioinformatics study confirmed significant relationships between pI and habitat, such as salinity and host environments, in prokaryotic proteomes, but it could not reveal significant correlations with oxygen and temperature requirements (Kiraga et al., 2007).

Notably, investigation of the relationship of genetic distance between bacterial strains and similarity of their theoretical 2D gels could not reveal a dependency on phylogeny (Knight et al., 2004). The most closely related organisms displayed very different proteome distributions as those typically observed between the organisms from different domains of life. Other study reported, based on analysis of pI distribution of 115 fully sequenced genomes, that the modal distributions do not reflect phylogeny or sequence evolution, but rather the chemical properties of amino acids (Weiller et al., 2004). Similarly, more recent investigation could not reveal any relation between pI bias and taxonomy both in prokaryotic and eukaryotic proteomes, however a phylogenetic signal was observed in mitochondrial proteomes (Kiraga et al., 2007). These findings are consistent with other observations that the pI values of protein orthologs are poorly conserved from species to species (Wilkins and Williams 1997; Nandi et al., 2005), further challenging the possibility of phylogenic pI adaptation to evolutionary constraints.

## Variation of Subcellular Localization-Specific pI Patterns

The proteome-wide relationships between protein pI and subcellular localization were analyzed in several bioinformatics studies of multiple proteomes. Initially, it was found that cytoplasmic proteins form the acidic modality and integral membrane proteins constitute the basic modality of the bimodal bacterial proteomes, whereas nuclear proteins may account for the third modality often observed in eukaryotes (Schwartz et al., 2001). Furthermore, it was demonstrated, using the experimental data of protein localization based on GFP tagging and microscopic detection of about 4,000 yeast proteins in 22 subcellular compartments, that the distributions of protein pI differ significantly in subcellular compartments (Huh et al., 2003; Ho et al., 2006). Although both the global and local intracellular pI values showed a bimodal distribution, the ratio between proteins of acidic and basic pI varied significantly among individual compartments. It was found that the proteomes of the cytoplasm, Golgi apparatus and vacuole are highly biased towards acidic pI, whereas the mitochondrial sub-proteome has a bias towards proteins of basic pI (Ho et al., 2006). Similarly, it was reported that yeast proteins localized in the organelles with alkaline pH, such as peroxisomes, endoplasmic reticulum and mitochondria, had relatively high pI values, whereas the proteins contained in the acidic organelles, such as vacuoles, Golgi and endosomes, tended to have rather low pIs (Brett et al., 2006). A detailed study of multiple proteomes from different biological species also confirmed that the proteomes of the cytoplasm, lysosomes, vacuoles and cytoskeleton are acidic, whereas those of mitochondria and the plasma membrane tend to be basic (Kiraga et al., 2007). Our recent study using one of the latest updates of human genome data disclosed a plethora of strong statistically significant correlations between protein pI and subcellular localization. Protein pI was found to correlate positively with mitochondrial and nuclear locations and negatively with lysosomal, cytoskeletal, peroxisomal and cytoplasmic ones (Kurotani et al., 2019, Figure 2). The most recent analysis of protein pI distributions in the interactomes across life domains has largely confirmed the above relationships between protein pI and subcellular localization (Chasapis and Konstantinoudis, 2020). The study also revealed that acidic proteins have the highest average number of interactions, whereas basic proteins have the lowest number of interactions in both prokaryotic and eukaryotic proteomes. A rationale behind these relationships remains unknown. Of note, the difference in the intracellular spatial distributions of proteins was proposed to be driven by a non-uniform distribution of intracellular pH (Baskin et al., 2006). This phenomenon based on the mechanism of pH-induced protein trapping was witnessed both in artificial systems and in living cells.

**FIGURE 2:**
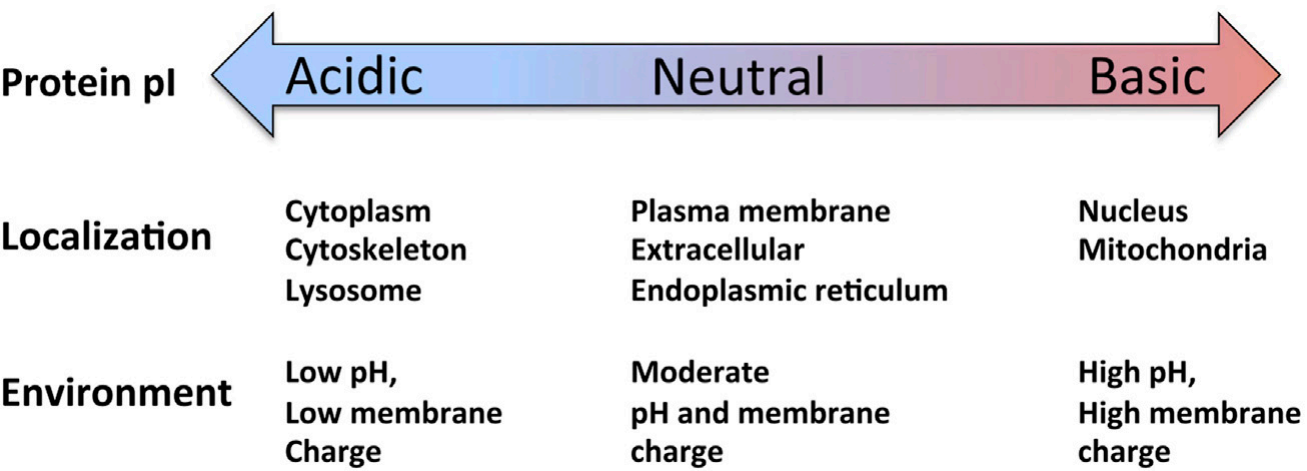
Relationships between protein pI and subcellular localization in the human proteome (see text for detailed explanations). Figure adapted from Kurotani et al., 2019 (CC BY 4 license, authors retain the copyright).

## Factors Behind the Variation of Subcellular pI Distributions

The variation of the localization-specific pI distributions was linked to the fact that local pH is different in subcellular compartments. It was reported that protein pIs averaged over a subcellular location correspond to experimentally measured intra-organellar pH in different compartments of the yeast cell and further speculated that subcellular protein pI and intra-organelle pH might have co-evolved to optimize protein function (Brett et al., 2006). However, this finding is difficult to reconcile with the notion that proteins are least soluble at the pH that corresponds to their pI. Indeed, a tendency has been observed for the averaged values of local pI distributions to differ from local pH (Chan et al., 2006; Chan and Warwicker, 2009). Furthermore, some analyses of multiple bacterial and eukaryotic proteomes failed to detect any statistically significant relationship between local pI distributions and subcellular intra-organelle pH (Wu et al., 2006; Kiraga et al., 2007).

On the other hand, it was reported that the folded states of proteins are often most stable at pH values near their pI, and these values also correlate with their optimal pH for function (Alexov, 2004; Talley and Alexov, 2010; Loell and Nanda, 2018). The evidence has been presented for adaptation of the protein pH dependence, rather than protein pI, to local subcellular pH. The average pH of maximal stability, but not the average pI of proteins in a subcellular compartment, was demonstrated to correlate with subcellular pH (Chan et al., 2006; Chan and Warwicker, 2009; Garcia-Moreno, 2009). In this connection, it was shown that the pH optimum for protein stability and activity can differ significantly from the pI value (Alexov, 2004; Talley and Alexov, 2010). The recent bioinformatics analysis of the human proteome confirmed that the specific pI distributions at different subcellular locations are governed by local physicochemical environment and further suggested that the local pH and organelle membrane charge are the main factors responsible for variation of the intracellular localization-specific pI profiles (Kurotani et al., 2019; see next section for details). Notably, the study failed to detect a statistically significant correlation between the mean values of local pI distributions and intra-organelle pH alone, however, it was observed that the proteins in alkaline compartments tended to have higher mean pI values than those in acidic organelles.

Furthermore, some bioinformatics studies addressed proteome-wide relationships between protein pI, intracellular localization and functional classification. Using the COG database, which lists gene orthologs present across completed genomes and assigns their functional classification, both the invariant and highly changeable proteins, which occur with a high frequency, have been identified in different regions of proteome-wide pI distributions (Nandi et al., 2005). In addition, a significant pI distribution bias, acidic or alkaline, was reported for certain protein functional classes localized in specific subcellular compartments (Wang and Tang, 2017).

## Generalized View of Localization-Specific pI Patterns (Importance of Local pH and Membrane Charge)

Thus, multiple bioinformatics studies converge on the assumption that the whole-proteome pI patterns adapt to environmental constraints and, in particular, the specific pI distribution at a certain subcellular location is defined by local environment. Our recent comprehensive analysis of 32,138 human proteins predicted to reside in 10 subcellular compartments, revealed the existence of strong relationships between protein pI and subcellular localization (Kurotani et al., 2019). Particularly, a robust positive correlation was witnessed between protein pI and propensity for mitochondrial and nuclear localization, and a negative correlation was observed for cytoskeletal, cytoplasmic, peroxisomal, lysosomal and endoplasmic reticulum proteins. These findings are broadly consistent with the data obtained by previous analyses of multiple prokaryotic and eukaryotic proteomes (Schwartz et al., 2001; Brett et al., 2006; Ho et al., 2006; Kiraga et al., 2007). The proteome-wide relationships between protein pI and subcellular localization are summarized in Figure 2.

Another important result of the study is the finding that organelle-specific protein pI patterns are physically defined by local pH and membrane charge. Relationships between the local subcellular pH and pI distributions have been explicitly addressed in previous studies; they are discussed in section 6 of the present paper. However, the effect of membrane charge on the pI patterns of local sub-proteomes has not been thoroughly scrutinized. Considering that the membrane composition and content of the negatively charged membrane lipids, such as phosphatidylserine and phosphatidylinositol, vary greatly in intracellular organelles, ranging from 2% in peroxisomes to more than 17% in nuclei and ER (Yang et al., 2003; Van Meer et al., 2008; Kurotani et al., 2019), the membrane charge could be regarded as a likely factor related to the variation of intracellular localization-specific patterns. Although the correlation between organelle membrane charge and mean local pI was not statistically significant, a composite function of the two variables, compartment pH and membrane charge, could approximate localization-specific mean pI with a statistically significant coefficient of determination (Kurotani et al., 2019). The result indicates that local pH and membrane charge jointly define intracellular localization-specific pI patterns. In a practical sense, the finding that membrane charge affects organelle-specific protein pI patterns can be useful when considering intracellular targeting of both endogenous and ectopically expressed exogenous proteins.

## Concluding Remarks

Genome sequencing has provided the information about all cellular and organismal proteins in many species. However, comprehension of life processes requires their further investigation at different levels. Uncovering subcellular localization of proteins with various physicochemical, structural and functional traits can reveal intracellular organization of proteomes and provide deeper understanding of their functioning. The recently disclosed relationships between protein pI and subcellular localization, as reviewed in this paper, contribute to spatial characterization of cellular processes. Still, the origin and mechanisms driving diversification of intracellular localization-specific pI patterns remain unknown. Although the possibility of positive evolutionary selection, which can promote beneficial protein pI patterns, seems unlikely (see section 4 for details), it was recently suggested that neutral evolution, i.e., accumulation of random mutations that have minimal impact on fitness and functional selection, might underline potential adjustment of protein pI to subcellular pH. It was revealed that the neutral evolutionary process leading to fixation of titratable residues in the protein core could likely be driven by marginal effects on protein stability (Loell and Nanda, 2018). Further proteomics and evolutionary studies are necessary to elucidate the factors that define subcellular localization of proteins with different physicochemical and functional traits.

## References

[B1] Alexov E. (2004). Numerical Calculations of the pH of Maximal Protein Stability. The Effect of the Sequence Composition and Three-Dimensional Structure. Eur. J. Biochem. 271 (1), 173–185. 10.1046/j.1432-1033.2003.03917.x 14686930

[B2] Arakawa T. Timasheff S. N. (1985). Theory of Protein Solubility. Methods Enzymol. 114, 49–77. 10.1016/0076-6879(85)14005-x 4079776

[B3] Audain E. Ramos Y. Hermjakob H. Flower D. R. Perez-Riverol Y. (2016). Accurate Estimation of Isoelectric point of Protein and Peptide Based on Amino Acid Sequences. Bioinformatics (Oxford, England) 32 (6), 821–827. 10.1093/bioinformatics/btv674 PMC5939969 26568629

[B4] Baskin E. M. Bukshpan S. Zilberstein G. V. (2006). pH-Induced Intracellular Protein Transport. Phys. Biol. 3 (2), 101–106. 10.1088/1478-3975/3/2/002 16829696

[B5] Bjellqvist B. Basse B. Olsen E. Celis J. E. (1994). Reference Points for Comparisons of Two-Dimensional Maps of Proteins from Different Human Cell Types Defined in a pH Scale where Isoelectric Points Correlate with Polypeptide Compositions. Electrophoresis 15 (3-4), 529–539. 10.1002/elps.1150150171 8055880

[B6] Bjellqvist B. Hughes G. J. Pasquali C. Paquet N. Ravier F. Sanchez J.-C. (1993). The Focusing Positions of Polypeptides in Immobilized pH Gradients Can Be Predicted from Their Amino Acid Sequences. Electrophoresis 14 (10), 1023–1031. 10.1002/elps.11501401163 8125050

[B7] Blattner F. R. Plunkett G. 3rd Bloch C. A. Perna N. T. Burland V. Riley M. (1997). The Complete Genome Sequence of *Escherichia C* K-12. Science 277 (5331), 1453–1462. 10.1126/science.277.5331.1453 9278503

[B8] Brett C. L. Donowitz M. Rao R. (2006). Does the Proteome Encode Organellar pH? FEBS Lett. 580 (3), 717–719. 10.1016/j.febslet.2005.12.103 16413548

[B9] Bunkute E. Cummins C. Crofts F. J. Bunce G. Nabney I. T. Flower D. R. (2015). PIP-DB: the Protein Isoelectric Point Database. Bioinformatics 31 (2), 295–296. 10.1093/bioinformatics/btu637 25252779

[B10] Cargile B. J. Bundy J. L. Freeman T. W. Stephenson J. L. Jr (2004). Gel Based Isoelectric Focusing of Peptides and the Utility of Isoelectric point in Protein Identification. J. Proteome Res. 3 (1), 112–119. 10.1021/pr0340431 14998171

[B11] Cargile B. J. Sevinsky J. R. Essader A. S. Eu J. P. Stephenson J. L. Jr (2008). Calculation of the Isoelectric point of Tryptic Peptides in the pH 3.5-4.5 Range Based on Adjacent Amino Acid Effects. Electrophoresis 29 (13), 2768–2778. 10.1002/elps.200700701 18615785

[B12] Carugo O. (2007). Isoelectric Points of Multi-Domain Proteins. Bioinformation 2 (3), 101–104. 10.6026/97320630002101 18292801 PMC2248714

[B13] Chan P. Lovrić J. Warwicker J. (2006). Subcellular pH and Predicted pH-Dependent Features of Proteins. Proteomics 6 (12), 3494–3501. 10.1002/pmic.200500534 16705750

[B14] Chan P. Warwicker J. (2009). Evidence for the Adaptation of Protein pH-Dependence to Subcellular pH. BMC Biol. 7, 69. 10.1186/1741-7007-7-69 19849832 PMC2770037

[B15] Chasapis C. T. Konstantinoudis G. (2020). Protein Isoelectric point Distribution in the Interactomes Across the Domains of Life. Biophysical Chem. 256, 106269. 10.1016/j.bpc.2019.106269 31733408

[B16] Garcia-Moreno B. (2009). Adaptations of Proteins to Cellular and Subcellular pH. J. Biol. 8 (11), 98. 10.1186/jbiol199 20017887 PMC2804283

[B17] Gasteiger E. Gattiker A. Hoogland C. Ivanyi I. Appel R. D. Bairoch A. (2003). ExPASy: The Proteomics Server for In-Depth Protein Knowledge and Analysis. Nucleic Acids Res. 31 (13), 3784–3788. 10.1093/nar/gkg563 12824418 PMC168970

[B18] Gauci S. van Breukelen B. Lemeer S. M. Krijgsveld J. Heck A. J. R. (2008). A Versatile Peptide pI Calculator for Phosphorylated and N-Terminal Acetylated Peptides Experimentally Tested Using Peptide Isoelectric Focusing. Proteomics 8 (23-24), 4898–4906. 10.1002/pmic.200800295 19003858

[B19] Ho E. Hayen A. Wilkins M. R. (2006). Characterisation of Organellar Proteomes: A Guide to Subcellular Proteomic Fractionation and Analysis. Proteomics 6 (21), 5746–5757. 10.1002/pmic.200600241 17068763

[B20] Hoogland C. Mostaguir K. Sanchez J.-C. Hochstrasser D. F. Appel R. D. (2004). SWISS-2DPAGE, Ten Years Later. Proteomics 4 (8), 2352–2356. 10.1002/pmic.200300830 15274128

[B21] Huh W.-K. Falvo J. V. Gerke L. C. Carroll A. S. Howson R. W. Weissman J. S. (2003). Global Analysis of Protein Localization in Budding Yeast. Nature 425 (6959), 686–691. 10.1038/nature02026 14562095

[B22] Kawashima T. Amano N. Koike H. Makino S.-i. Higuchi S. Kawashima-Ohya Y. (2000). Archaeal Adaptation to Higher Temperatures Revealed by Genomic Sequence of Thermoplasma Volcanium. Proc. Natl. Acad. Sci. 97 (26), 14257–14262. 10.1073/pnas.97.26.14257 11121031 PMC18905

[B23] Kiraga J. Mackiewicz P. Mackiewicz D. Kowalczuk M. Biecek P. Polak N. (2007). The Relationships between the Isoelectric Point and: Length of Proteins, Taxonomy and Ecology of Organisms. BMC genomics 8, 163. 10.1186/1471-2164-8-163 17565672 PMC1905920

[B24] Knight C. G. Kassen R. Hebestreit H. Rainey P. B. (2004). From the Cover: Global Analysis of Predicted Proteomes: Functional Adaptation of Physical Properties. Proc. Natl. Acad. Sci. 101 (22), 8390–8395. 10.1073/pnas.0307270101 15150418 PMC420404

[B25] Kozlowski L. P. (2017). Proteome-pI: Proteome Isoelectric Point Database. Nucleic Acids Res. 45 (D1), D1112–D1116. 10.1093/nar/gkw978 27789699 PMC5210655

[B26] Kurotani A. Takagi T. Toyama M. Shirouzu M. Yokoyama S. Fukami Y. (2010). Comprehensive Bioinformatics Analysis of Cell‐Free Protein Synthesis: Identification of Multiple Protein Properties that Correlate with Successful Expression. FASEB j. 24 (4), 1095–1104. 10.1096/fj.09-139527 19940260

[B27] Kurotani A. Tokmakov A. A. Sato K.-I. Stefanov V. E. Yamada Y. Sakurai T. (2019). Localization-Specific Distributions of Protein pI in Human Proteome Are Governed by Local pH and Membrane Charge. BMC Mol. Cel Biol 20 (1), 36. 10.1186/s12860-019-0221-4 PMC6701068 31429701

[B28] Link A. J. Robison K. Church G. M. (1997). Comparing the Predicted and Observed Properties of Proteins Encoded in the Genome ofEscherichia Coli K-12. Electrophoresis 18 (8), 1259–1313. 10.1002/elps.1150180807 9298646

[B29] Loell K. Nanda V. (2018). Marginal Protein Stability Drives Subcellular Proteome Isoelectric point. Proc. Natl. Acad. Sci. USA 115 (46), 11778–11783. 10.1073/pnas.1809098115 30385634 PMC6243250

[B30] Maldonado A. A. Ribeiro J. M. Sillero A. (2010). Isoelectric point, Electric Charge, and Nomenclature of the Acid-Base Residues of Proteins. Biochem. Mol. Biol. Educ. 38 (4), 230–237. 10.1002/bmb.20405 21567833

[B31] Medjahed D. Smythers G. W. Powell D. A. Stephens R. M. Lemkin P. F. Munroe D. J. (2003). VIRTUAL2D: A Web-Accessible Predictive Database for Proteomics Analysis. Proteomics 3 (2), 129–138. 10.1002/pmic.200390021 12601805

[B32] Nandi S. Mehra N. Lynn A. M. Bhattacharya A. (2005). Comparison of Theoretical Proteomes: Identification of COGs with Conserved and Variable pI within the Multimodal pI Distribution. BMC Genomics 6, 116. 10.1186/1471-2164-6-116 16150155 PMC1249567

[B33] Russell S. T. Warshel A. (1985). Calculations of Electrostatic Energies in proteinsThe Energetics of Ionized Groups in Bovine Pancreatic Trypsin Inhibitor. J. Mol. Biol. 185 (2), 389–404. 10.1016/0022-2836(85)90411-5 2414450

[B34] Schwartz R. Ting C. S. King J. (2001). Whole Proteome pI Values Correlate with Subcellular Localizations of Proteins for Organisms within the Three Domains of Life. Genome Res. 11 (5), 703–709. 10.1101/gr.gr-1587r 11337469

[B35] Sillero A. Ribeiro J. M. (1989). Isoelectric Points of Proteins: Theoretical Determination. Anal. Biochem. 179 (2), 319–325. 10.1016/0003-2697(89)90136-x 2774179

[B36] Srivastava D. Santiso E. Gubbins K. Barroso da Silva F. L. (2017). Computationally Mapping pKa Shifts Due to the Presence of a Polyelectrolyte Chain Around Whey Proteins. Langmuir 33 (42), 11417–11428. 10.1021/acs.langmuir.7b02271 28859478

[B37] Talley K. Alexov E. (2010). On the pH-Optimum of Activity and Stability of Proteins. Proteins 78 (12), 2699–2706. 10.1002/prot.22786 20589630 PMC2911520

[B38] Tokmakov A. A. Kurotani A. Shirouzu M. Fukami Y. Yokoyama S. (2014). Bioinformatics Analysis and Optimization of Cell-Free Protein Synthesis. Methods Mol. Biol. (Clifton, N.J.) 1118, 17–33. 10.1007/978-1-62703-782-2_2 24395407

[B39] Urquhart B. L. Atsalos T. E. Roach D. Basseal D. J. Bjellqvist B. Britton W. L. (1997). 'Proteomic Contigs' ofMycobacterium Tuberculosis andMycobacterium Bovis (BCG) Using Novel Immobilised pH Gradients. Electrophoresis 18 (8), 1384–1392. 10.1002/elps.1150180813 9298652

[B40] Van Meer G. Voelker D. R. Feigenson G. W. (2008). Membrane Lipids: Where They Are and How They Behave. Nat. Rev. Mol. Cel Biol 9 (2), 112–124. 10.1038/nrm2330 PMC2642958 18216768

[B41] VanBogelen R. A. Schiller E. E. Thomas J. D. Neidhardt F. C. (1999). Diagnosis of Cellular States of Microbial Organisms Using Proteomics. Electrophoresis 20 (11), 2149–2159. 10.1002/(sici)1522-2683(19990801)20:11<2149:aid-elps2149>3.0.co;2-n 10493120

[B42] Wang T. Tang H. (2017). The Physical Characteristics of Human Proteins in Different Biological Functions. PloS one 12 (5), e0176234. 10.1371/journal.pone.0176234 28459865 PMC5411090

[B43] Weiller G. F. Caraux G. Sylvester N. (2004). The Modal Distribution of Protein Isoelectric Points Reflects Amino Acid Properties Rather Than Sequence Evolution. Proteomics 4 (4), 943–949. 10.1002/pmic.200200648 15048976

[B44] Wilkins M. R. Williams K. L. (1997). Cross-Species Protein Identification Using Amino Acid Composition, Peptide Mass Fingerprinting, Isoelectric point and Molecular Mass: A Theoretical Evaluation. J. Theor. Biol. 186 (1), 7–15. 10.1006/jtbi.1996.0346 9176634

[B45] Wu S. Wan P. Li J. Li D. Zhu Y. He F. (2006). Multi-Modality of pI Distribution in Whole Proteome. Proteomics 6 (2), 449–455. 10.1002/pmic.200500221 16317776

[B46] Yang J. Han X. Gross R. W. (2003). Identification of Hepatic Peroxisomal Phospholipase A(2) and Characterization of Arachidonic Acid-Containing Choline Glycerophospholipids in Hepatic Peroxisomes. FEBS Lett. 546 (2-3), 247–250. 10.1016/s0014-5793(03)00581-7 12832049

